# Scotch Humor

**Published:** 1874-04

**Authors:** 


Scotch Humor.— Here are a couple of specimens of it: "How," said an Aberdeen philosopher to some young students he was examining, "how, for instance, do astronomers measure the distance to the sun?" It rather posed the class, till a young Shetlander, with sandy hair, exclaimed: "They calculate one fourth of the distance, and then multiply by four." An Aberdeen lad with a very good appetite swallowed a small leaden bullet. His friends were very much alarmed about it. The doctor was found, heard the dismal tale, and with as much unconcern as he would manifest in a case of common headache, wrote the following laconic note to the lad's father: "Don't alarm yourself. If, after three weeks, the bullet is not removed, give the boy a charge of powder. P. S.— Don't shoot the boy at anybody."

